# Geranylgeranyltransferase I is essential for dendritic development of cerebellar Purkinje cells

**DOI:** 10.1186/1756-6606-3-18

**Published:** 2010-06-11

**Authors:** Kong-Yan Wu, Xiu-Ping Zhou, Zhen-Ge Luo

**Affiliations:** 1 Institute of Neuroscience, State Key Laboratory of Neuroscience, Shanghai Institutes for Biological Sciences, Chinese Academy of Sciences, 320 Yue Yang Road, Shanghai 200031, China; 2 Department of Neurosurgery, Xuzhou Medical College, 84 West Huai-hai Road, Xuzhou, Jiangsu, 221002, China

## Abstract

**Background:**

During cerebellar development, Purkinje cells (PCs) form the most elaborate dendritic trees among neurons in the brain, but the mechanism regulating PC arborization remains largely unknown. Geranylgeranyltransferase I (GGT) is a prenyltransferase that is responsible for lipid modification of several signaling proteins, such as Rho family small GTPase Rac1, which has been shown to be involved in neuronal morphogenesis. Here we show that GGT plays an important role in dendritic development of PCs.

**Results:**

We found that GGT was abundantly expressed in the developing rat cerebellum, in particular molecular layer (ML), the region enriched with PC dendrites. Inhibition or down-regulation of GGT using small interference RNA (siRNA) inhibited dendritic development of PCs. In contrast, up-regulation of GGT promoted dendritic arborization of PCs. Furthermore, neuronal depolarization induced by high K^+ ^or treatment with brain-derived neurotrophic factor (BDNF) promoted membrane association of Rac1 and dendritic development of PCs in cultured cerebellar slices. The effect of BDNF or high K^+ ^was inhibited by inhibition or down-regulation of GGT.

**Conclusion:**

Our results indicate that GGT plays an important role in Purkinje cell development, and suggest a novel role of GGT in neuronal morphogenesis *in vivo*.

## Background

Protein prenyltransferases, mainly farnesyl transferase (FT) and geranylgeranyl transferase I (GGT), are responsible for posttranslational lipidation of proteins with C-terminal "CAAX" motifs, where C is cysteine, A is often an aliphatic amino acid, and X at the C-terminus determines the specificity of protein prenylation [1,2]. GGT catalyzes the transfer of a 20-carbon prenyl group from geranylgeranyl pyrophosphate (GGPP) to a cysteine residue of proteins usually with leucine or phenylalanine at their C-terminus [1,3]. GGT substrates include K-Ras and Rho family small GTPases, such as Rac1, Cdc42 and RhoA, whose prenylation is essential for membrane localization, activation, and functions in various signaling pathways [4]. Given that mutations of these small GTPases are oncogenic to cause malignant transformation, many studies have tried to use GGT inhibitors to suppress tumor growth [5-8]. A recent report shows that gene ablation of GGTase I-specific beta subunit (GGTβ) reduced lung tumor formation, probably by eliminating GGTase activity, disrupting the actin cytoskeleton, reducing cell migration, and/or blocking tumor cell proliferation [9]. Thus GGT is a potential target for anti-cancer drug development.

Interestingly, the highest activity of GGT is often observed in the brain [10]. Indeed, bovine brain has been used as a rich source for GGT purification [3]. However, the role of GGT in neuronal system is poorly understood. Our previous study shows that GGT is localized at the neuromuscular junction and regulates agrin-induced clustering of acetylcholine receptors (AChR) by interacting with muscle specific receptor tyrosine kinase (MuSK) [11]. Recently, we found that neuronal activity and BDNF activate GGT, which in turn promotes membrane recruitment of Rac1 and increases dendritic arborization of cultured hippocampal neurons [12]. Furthermore, the activity of GGT in the rat hippocampus markedly increased when rats were put in a novel environment [12]. Thus, GGT plays an important role in neuronal development. Nevertheless, the function of GGT in regulating neural development needs further investigation, especially in more intact systems.

During postnatal cerebellar development, Purkinje cells (PCs) form the most elaborate dendritic trees among neurons in the brain, however the mechanism governing dendrite development of PCs has not been completely understood [13]. Here we demonstrate a role of GGT in the morphogenesis of PCs in cultured cerebellar slices. We found that GGT is enriched in the molecular layer of developing cerebellum. Down-regulation of GGT markedly affected dendritic arborization of PCs. In agreement with the notion that BDNF or neuronal activity activates GGT [12], we found that the enhancement effect of BDNF or high K^+ ^on dendrite development of PCs was dramatically impeded by down-regulation of GGT. Thus GGT plays an important role in cerebellar neuron development.

## Results

### Expression of GGT in the rat cerebellum

**Figure 1:**
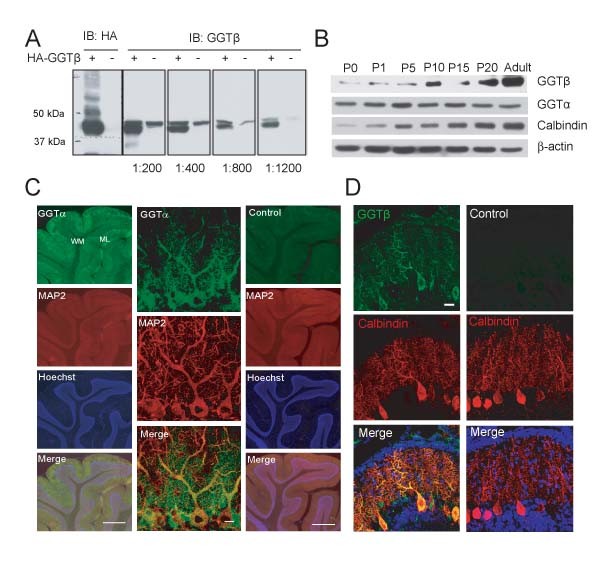
**Expression and localization of GGT in the rat cerebellum**. A) Lysates of HEK293 cells transfected with HA-GGTβ or vehicle plasmid were subjected to immunoblotting (IB) with anti-HA or -GGTβ antibody at indicated dilution. B) Homogenates of cerebellum from rats at indicated ages were subjected to IB with indicated antibodies. C) P10 rat cerebellar sections were stained with antibody against GGTα or control antibody, together with MAP2. Hoechst was used to mark cell layers. ML: molecular layer; WM: white matter. Scale bar is 200 μm (left and right lanes) or 20 μm (middle lane). D) P15 rat cerebellar sections were stained with antibody against GGTβ or control antibody, together with Calbindin. DAPI was used to mark cell layers. Scale bar is 20 μm.

Given that GGTα is shared between GGT and FT [1], to determine the expression of GGT in the brain, we generated an antibody, which was raised against synthetic GGTβ peptide. As shown in Figure 1A, this antibody recognized bands corresponding to exogenous HA-rGGTβ (rat GGTβ), as well as endogenous hGGTβ (human GGTβ) expressed in HEK293 cells, respectively. Next, we determined the levels of GGT in the rat cerebellum at different stages. We found that cerebellar GGTβ progressively increased after birth, and this pattern is similar to that of Calbindin, an intracellular calcium binding protein which is often used as a marker for cerebellum Purkinje cells. However, levels of GGTα remained unchanged (Figure 1B). Next, we examined regional distribution of GGT in the rat cerebellum. We found that GGTα was highly expressed in the cerebellar molecular layer, the region enriched with Purkinje cell dendritic trees (Figure 1C, left lane). Particularly, GGTα was enriched in the cell bodies and dendritic arbors, which were stained positive for MAP2 (Figure 1C, middle lane). GGTβ was also found to be expressed in Purkinje cells (PCs) that were positively labeled with Calbindin antibody (Figure 1D). These results suggest that GGT might be involved in Purkinje cell development.

### Up-regulation of GGT promotes Purkinje cell arborization

**Figure 2:**
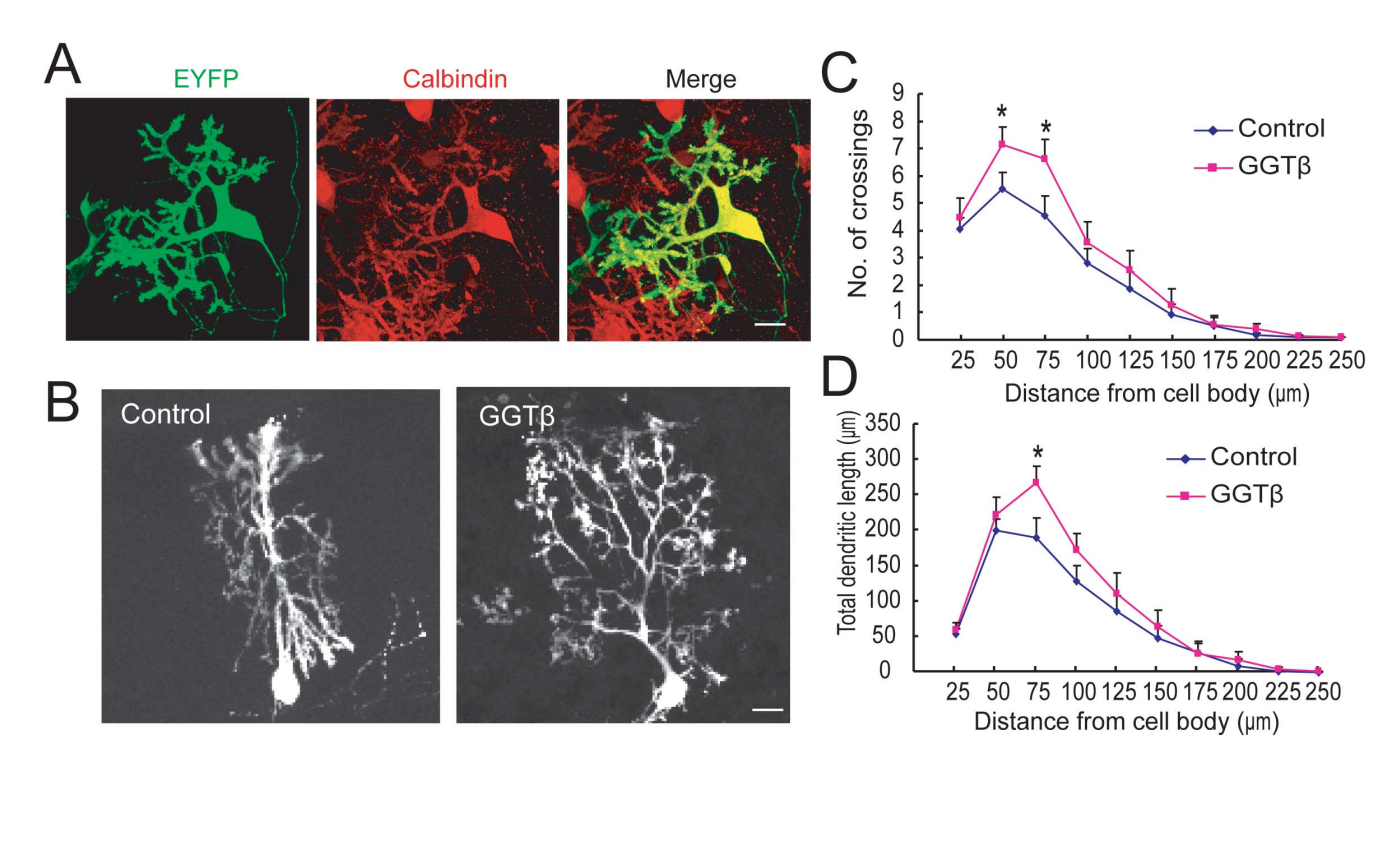
**Up-regulation of GGT promotes dendrite growth and branching of PCs**. Cerebellar slices of P11 rats were cultured for 2 days (DIV2) before transfection with pCAG-EYFP either alone or together with HA-GGTβ at the ratio of 1:3, followed by staining with anti-Calbindin antibody at DIV5. A) Shown is a representative PC expressing YFP, which is positively stained with Calbinin antibody. B) Representative images of HA-GGTβ or vehicle plasmid-transfected PCs. C) The number of crossings between PC dendrites and circles at indicated distance from soma is quantified to reflect dendritic branches. D) Total dendrite length between neighboring circles at the interval of 25 μm from soma is quantified. Data are shown as means ± SEM (n = 28 for control; n = 27 for HA-GGTβ). *P < 0.05. Student's *t *test. Scale bar is 20 μm.

Having determined the expression of GGT in the cerebellum, we next used gain-of function approach to examine the role of GGT in Purkinje cell dendrite development by over-expressing GGTβ. We prepared cerebellar slices from P11 rats and cultured as described previously [14,15]. Cerebellar slices were subjected to transfection with vehicle control or together with testing plasmid by a biolistic method, and the identity of Purkinje cells was revealed by positive staining of Calbindin, with co-transfected EYFP plasmid to mark transfected cells (Figure 2A). Alterations in the dendritic growth of transfected PCs were quantified by the Sholl analysis [16], which determines the number of crossings between dendritic branches and circles centered at the soma, reflecting the number of dendritic branches. We found that over-expression of HA-GGTβ caused an increase in the branching of PCs (Figure. 2B and 2C). The promoting effect on number of crossings was most remarkable at the distance of 50 or 75 μm to the cell body, where PCs branch extensively to form secondary and tertiary dendrites (Figure 2B and 2C). In addition, the total dendritic length between the circle of 50 and 75 μm to the cell body, was significantly increased in HA-GGTβ-transfected neurons (Figure 2D, P < 0.05). These results indicate that GGT promotes dendritic morphogenesis of Purkinje cells.

### GGT is required for dendritic growth and arborization of Purkinje cells in cultured cerebellar slices

**Figure 3:**
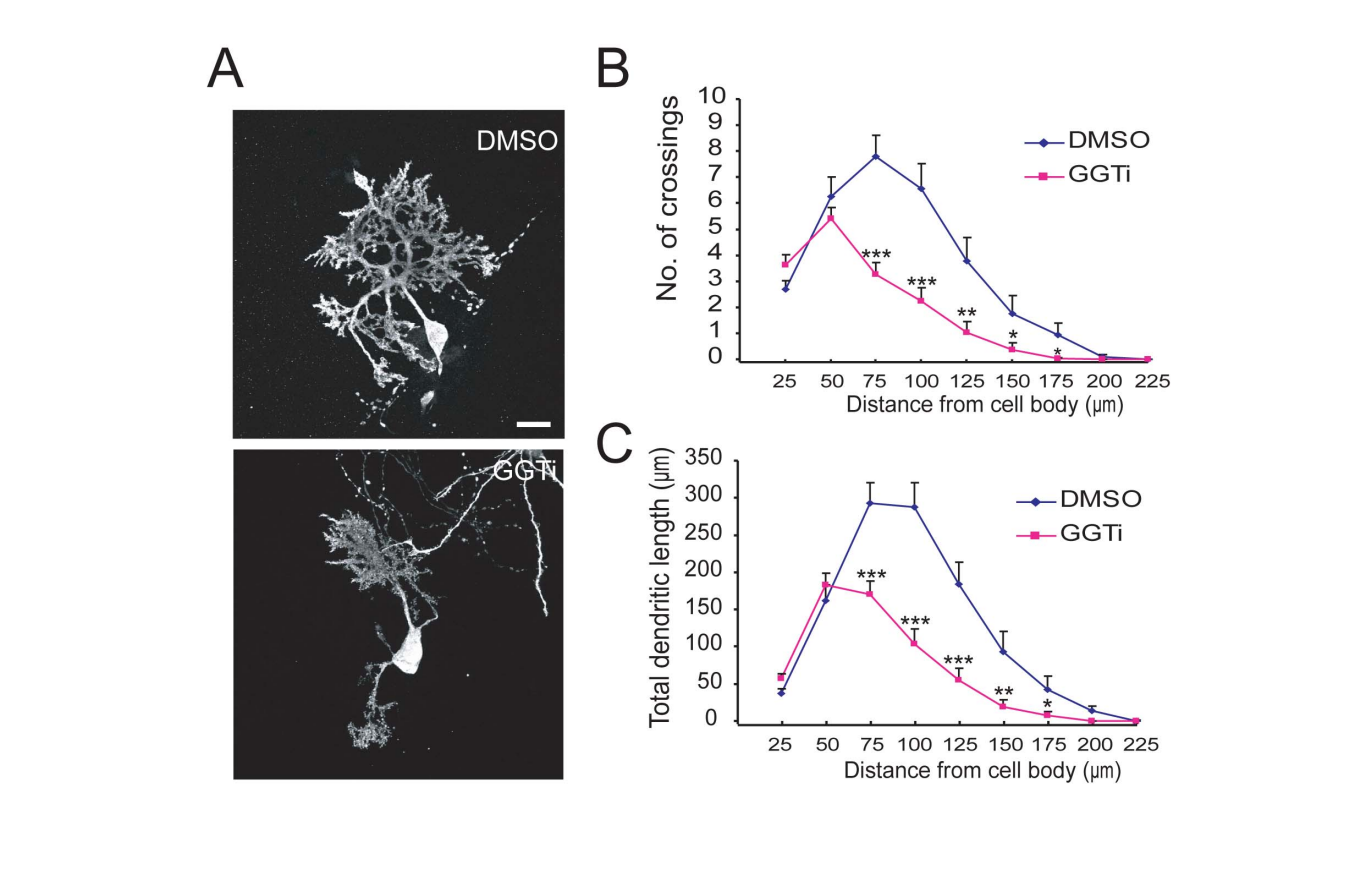
**Inhibition of GGT impedes dendrite development of PCs**. P11 rat cerebellar slices at DIV2 were transfected with pCAG-EYFP plasmid. At DIV3, cultured slices were treated with DMSO or GGTi-2147 (2.5 μM) for additional 3 days. A) Representative images of PCs at DIV5, after treatment with DMSO or GGTi-2147. B) Quantification for the number of crossings. C) Quantification for total dendritic length between neighboring circles at indicated distance from soma. Data are shown as means ± SEM (n = 31 for control; n = 31 for GGTi-2147). *P < 0.05, **P < 0.01, ***P < 0.001; Student's *t *test. Scale bar = 20 μm.

To further determine the role of GGT-mediated prenylation in PC arborization, we treated cultured cerebellar slices with GGT inhibitor GGTi-2147. We found that treatment with GGTi caused a marked decrease in the branching and growth of dendrites of PCs (Figure 3A-C). The inhibitory effect was reflected from decreased number of crossings between dendrites and circles with various distances from soma (Figure 3B) and total dendritic lengths between neighboring circles at the interval of 25 μm, e.g., 50 to 75 μm, 75 to 100 μm, 100 to 125 μm, etc (Figure 3C). Thus, GGT-mediated protein prenylation plays an important role in the morphogenesis of PCs.

**Figure 4:**
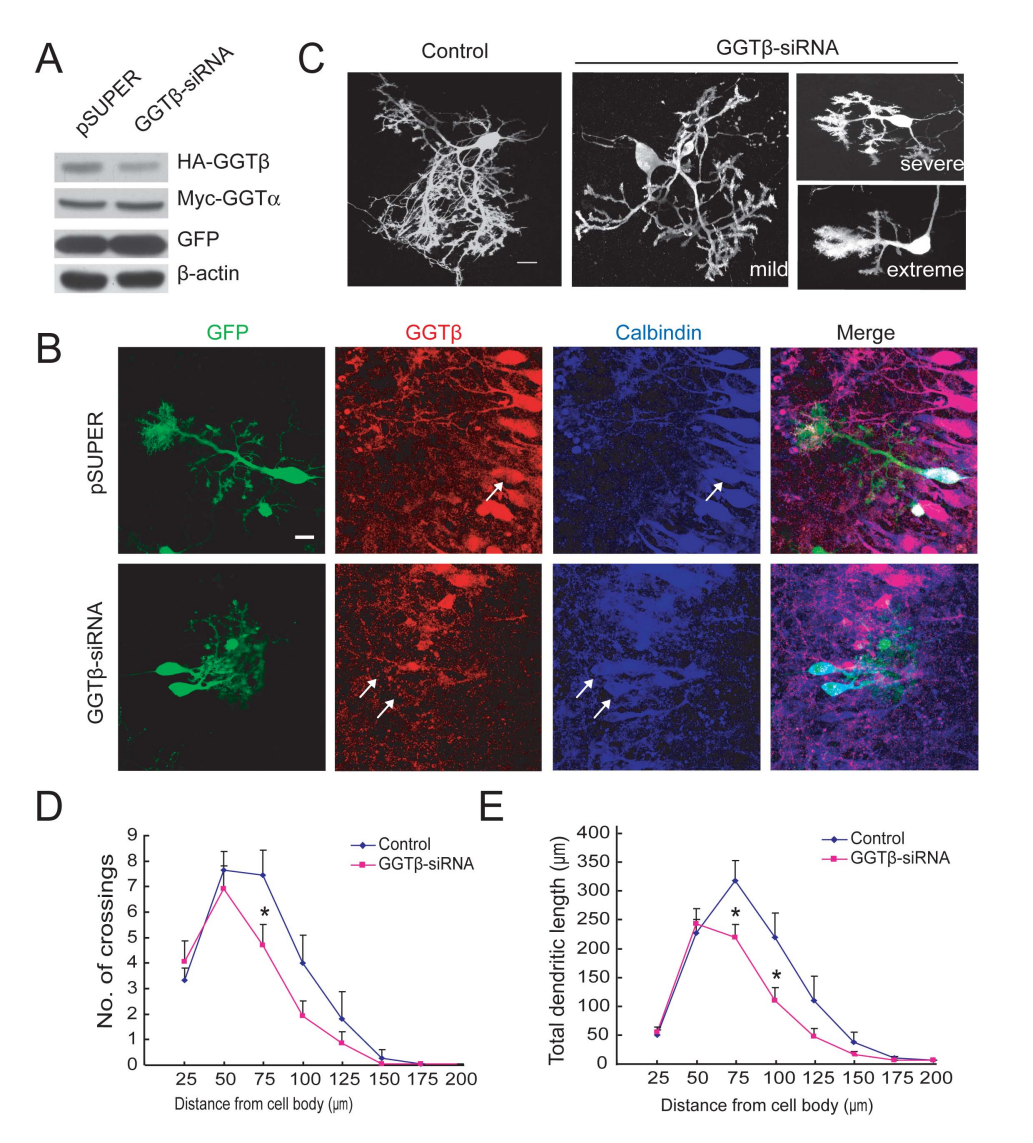
**Down regulation of GGTβ decreases dendrite growth and branching of PCs**. A) HEK293 cells were co-transfected with Myc-GGTα and HA-GGTβ, together with pSUPER-GGTβ-siRNA or pSUPER. Cell lysates were subjected to IB with HA, Myc, GFP, or β-actin antibodies. B) Cultured cerebellar slices were transfected with pSUPER or pSUPER-GGTβ-siRNA, followed by staining with antibodies against GGTβ and Calbindin. Note the decrease of GGTβ signals in GGTβ-siRNA -transfected PCs. Scale bar = 20 μm. C) Cultured cerebellar slices at DIV2 were transfected with pSUPER-GGTβ-siRNA or pSUPER, together with pCAG-EYFP plasmid (3:1) to mark transfected cells. Shown are representative images at DIV5. Scale bar = 20 μm. D) Quantification for the number of crossings between dendrites and circles with a radius of indicated distance. E) Quantification for total dendritic length in indicated fields. Data shown are presented as means ± SEM (n = 17 for control, n = 23 for GGTβ-siRNA). *P < 0.05, Student's *t *test. Scale bar is 20 μm.

Given the potential side effects of synthetic inhibitors, we took advantage of vector-based small interference RNA (siRNA) against GGTβ that has been shown to be able to down-regulate expression of endogenous GGTβ in primary neurons [12]. As shown in Figure 4A, this GGTβ-siRNA suppressed the expression of ectopic GGTβ, but not GGTα, expressed in HEK293 cells. The effectiveness of GGTβ-siRNA in suppressing endogenous GGTβ expression was observed in PCs of cultured cerebellar slices (Figure 4B). We found that the Purkinje cells developed much simpler dendritic arbors when transfected with GGT-siRNA, in comparison to control (Figure 4B). Transfected Purkinje cells were classified into three groups based on the severity of the phenotypes (mild: total dendritic length: >1000 μm; severe: 500-1000 μm; extreme: <500 μm) [17]. Among 23 GGT-siRNA-transfected neurons, the distribution of the phenotypes were 2 mild, 13 severe and 8 extreme (Figure 4C). Quantitatively, the number of crossings between dendrites and a 75 μm radius circle was significantly reduced in GGTβ-siRNA-transfected neurons, compared to control cells (Figure 4D, n = 17 for control, n = 23 for GGT-siRNA, P < 0.05). In addition, the total dendritic length between 50 and 75 μm or 75 and 100 μm circles to the soma, was significantly reduced in GGTβ-siRNA-transfected neurons (Figure 4E, P < 0.05). These results indicate that GGT itself plays a critical role in dendritic morphogenesis of PCs.

**Figure 5:**
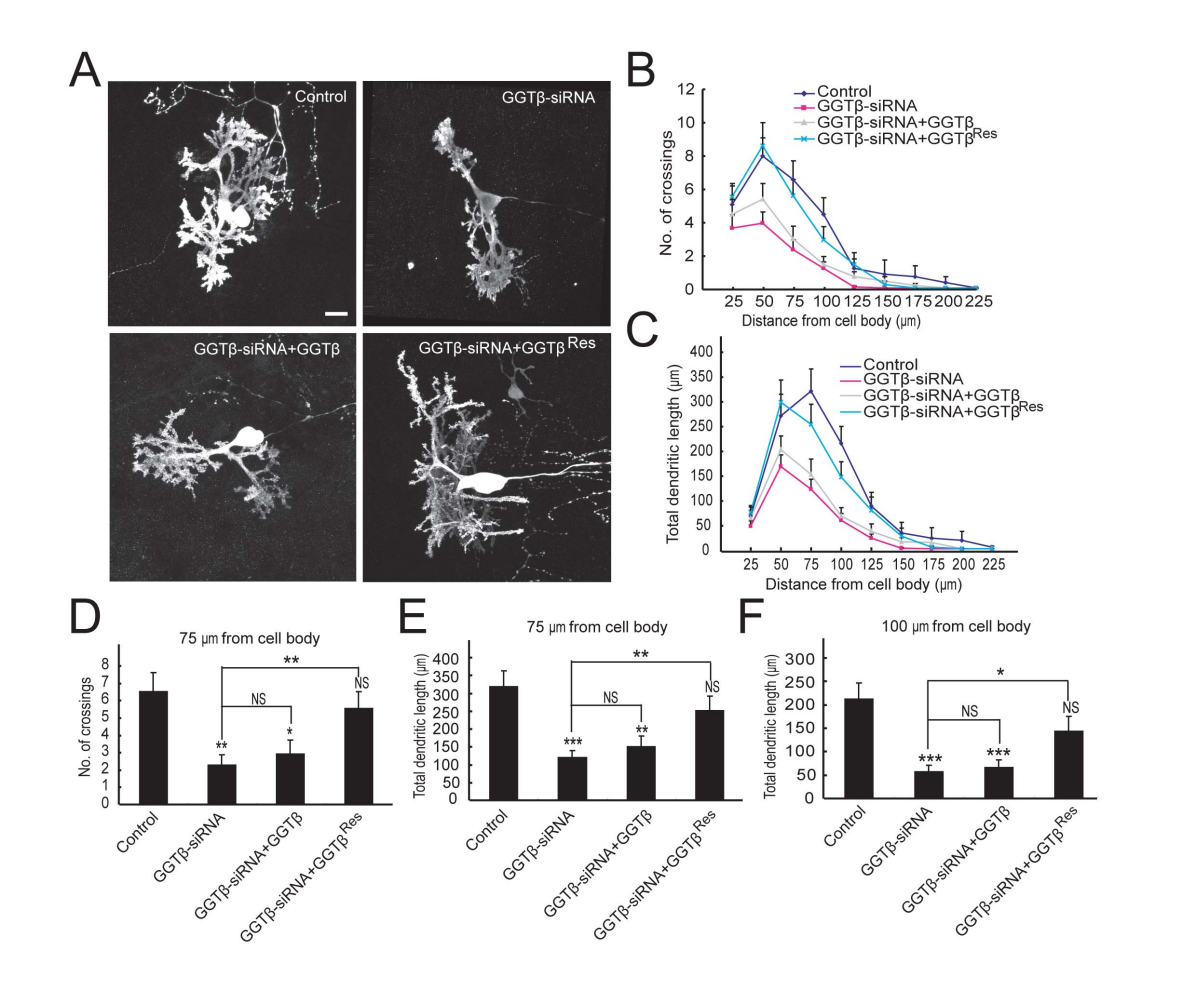
**Rescue effect of siRNA-resistant form of GGTβ**. A) Representative images of Purkinje cells after transfection with pSUPER (control), pSUPER-GGTβ-siRNA, or GGTβ-siRNA together with HA-GGTβ or HA-GGTβ^Res^. B) Quantification for the number of crossings. C) Quantification for total dendritic length between neighboring circles. D) Number of crossings at the circle with radius of 75 μm was quantitatively analyzed. E, F) Total dendritic length between neighboring circles at the interval of 25 μm (between 50 and 75 μm or between 75 and 100 μm). Data are shown as means ± SEM (n = 12 for control; n = 17 for GGT-siRNA; n = 15 for GGT-siRNA plus HA-GGTβ; n = 19 for GGT-siRNA plus HA-GGTβ^Res^). N.S. *P *> 0.05; *P < 0.05; **P < 0.01; ***P < 0.001. Student's *t *test. Scale bar = 20 μm.

To exclude possible off-target effect of GGTβ-siRNA, we performed rescue experiment with GGTβ^Res^, the siRNA-resistant form of GGTβ, which has been described in our previous report [12]. Cerebellar slices were transfected with GGTβ-siRNA either alone or together with HA-GGTβ or HA-GGTβ^Res^. We found that expression of HA-GGTβ^Res ^rescued dendritic development in GGTβ-siRNA-transfected neurons, whereas transfection with HA-GGTβ had no rescue effect (Figure 5A). The rescue effect could be reflected from the changes of the number of crossings (Figure 5B and 5D) and total dendritic length (Figure 5C, E, F) in indicated dendritic fields. These results suggest GGT effect on PC dendrtic development is specific.

### GGT is required for BDNF-mediated dendritic growth of Purkinje cells

**Figure 6:**
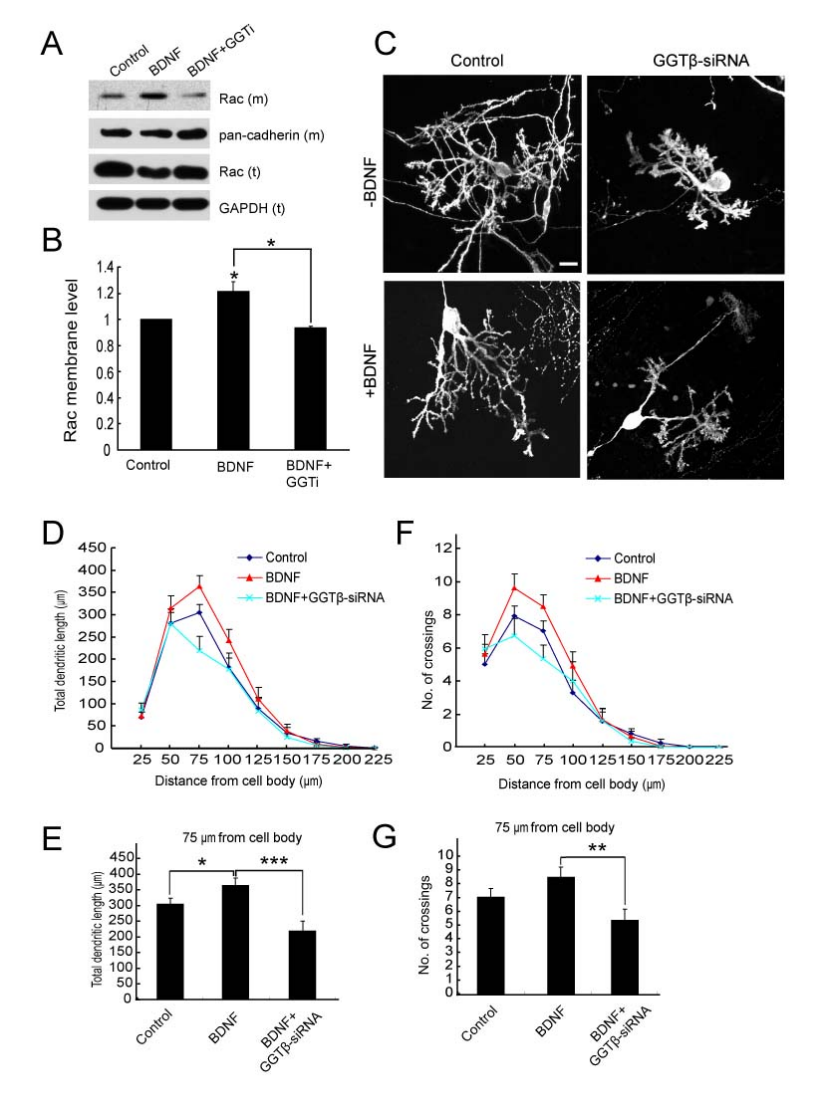
**Effect of GGT-siRNA on BDNF-induced Purkinje cell dendrite development**. A) Rat cerebellar slices at DIV2 were pre-treated with GGTi-2147 (2.5 μM) or vehicle (DMSO) for 45 min, followed by the treatment with BDNF (50 ng/ml) for 2 hr. Membrane fractions were separated and subjected to IB with antibodies against Rac and pan-cadherin. Total Rac or GAPDH was probed as loading controls. B) Quantification for the levels of membrane Rac, Rac (m). Data were shown as means ± SEM from three independent experiments. *P < 0.05. Student's *t *test. C) Representative images of Purkinje cells after transfection with pSUPER or pSUPER-GGTβ-siRNA, without or with 50 ng/ml BDNF treatments. D) Quantification for total dendritic length between neighboring circles. E) Quantification for total dendritic length between the circle of 50 and 75 μm. F) Quantification for the number of crossings at indicated distances from soma. G) Number of crossings at the circle with radius of 75 μm was quantitatively analyzed. Data are shown as means ± SEM (n = 33 for control; n = 44 for GGT-siRNA; n = 23 for BDNF; n = 21 for BDNF with GGT-siRNA). N.S. *P *> 0.05; *P < 0.05; **P < 0.01; ***P < 0.001. Student's *t *test. Scale bar = 20 μm.

Neurotrophins such as NGF, BDNF, NT-3, 4 are known to regulate dendritic growth [18-20]. Our previous report shows that GGT is activated by BDNF and mediates BDNF-induced dendrite growth [12]. In BDNF-knockout mice, growth of Purkinje cell dendrites is impeded [21]. Granule cell death was also observed in the BDNF-knockout mice, suggesting that the effect on Purkinje cell development could be indirect [21]. We determined the role of BDNF by treating cultured cerebellar slices with BDNF. We found that BDNF treatment caused an increase in the level of membrane-associated Rac, thereafter referred to as Rac (m) (Figure 6A and 6B), but had no effect on GGT expression (data not shown), suggesting the activation of GGT. This elevation is similar to that seen in cultured cortical neurons [12], and depended on the GGT activity, since the presence of GGTi-2147 prevented the increase in the level of Rac (m) induced by BDNF (Figure 6A and 6B). Furthermore, treatment with BDNF promoted dendritic growth, as reflected from increased total dendritic length between circles of 50 and 75 μm to the soma (Figure 6C-E). However, number of crossings was mildly, but not significantly, affected by the treatment with BDNF (Figure 6F and 6G). Thus, in our experimental conditions, BDNF stimulates Purkinje cell dendrite growth rather than arborization. The effect of BDNF on dendrite length was attenuated when endogenous GGT was down-regulated by GGTβ-siRNA (Figure 6D-G). This result suggests that GGT also participates in BDNF-mediated PC dendrite development.

### High K^+^-induced dendritic morphogenesis of Purkinje cells requires the function of GGT

**Figure 7:**
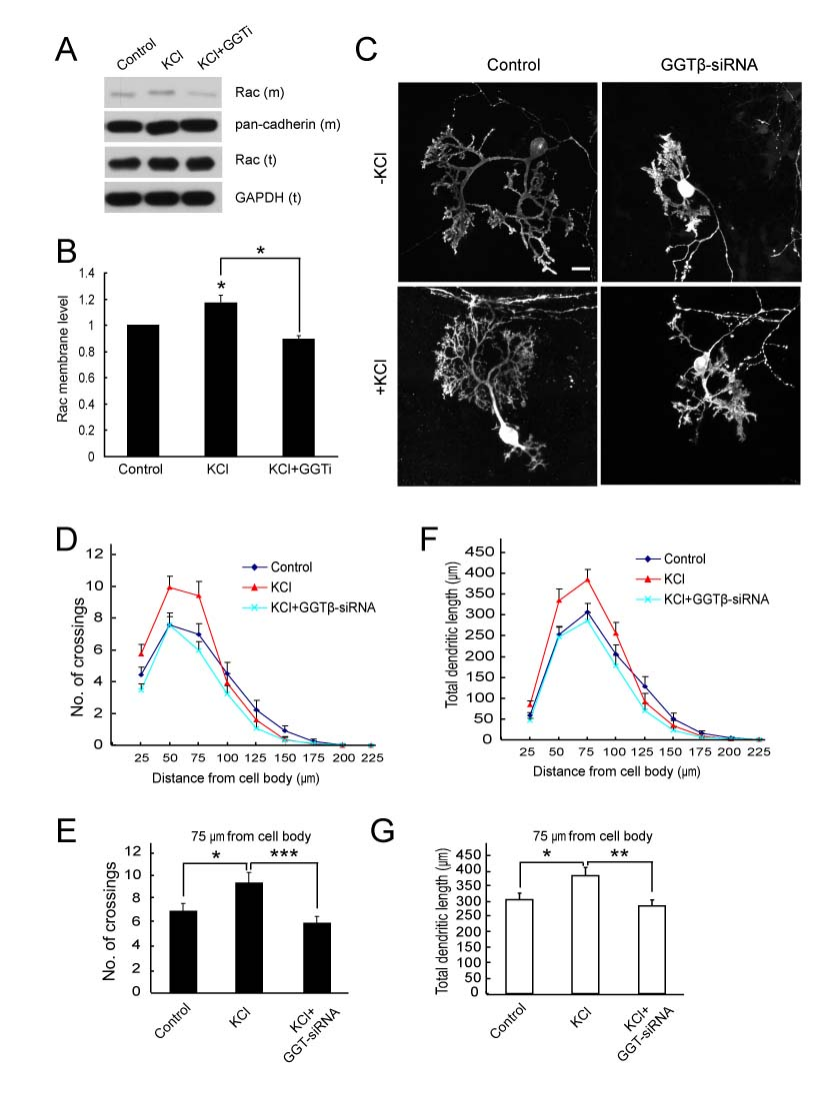
**Effect of GGT-siRNA on high K+-induced Purkinje cell dendrite development**. A) Rat cerebellar slices at DIV2 were pre-treated with GGTi-2147 (2.5 μM) or vehicle DMSO for 45 min, followed by the treatment with KCl (10 mM) for 2 hr. Membrane fractions were separated and subjected to IB with indicated antibodies. B) Quantification for the levels of membrane Rac, Rac (m). Data were shown as means ± SEM from three independent experiments. *P < 0.05. Student's *t *test. C) Representative images of Purkinje cells transfected with pSUPER or pSUPER-GGTβ-siRNA, without or with 10 mM KCl treatments. D, E) Quantification for the number of crossings at indicated distances from soma. F) Quantification for total dendritic length between neighboring circles. G) Total dendritic length between the circle of 50 and 75 μm. Data are shown as means ± SEM (n = 39 for control; n = 47 for GGT-siRNA; n = 36 for KCl; n = 34 for KCl with GGT-siRNA). N.S. *P *> 0.05; *P < 0.05; **P < 0.01; ***P < 0.001. Student's *t *test. Scale bar = 20 μm.

Like other types of neurons, dendritic differentiation of PCs is also shown to be regulated by neuronal activity [22]. Our previous study shows that high K^+^-induced depolarization promotes dendrite development of cultured hippocampal neurons, and this process requires TrkB-mediated activation of GGT [12]. In line with this notion, exposure of cultured cerebellar slices to high K^+ ^resulted in an increase in the level of Rac (m), but not total Rac or GGT (Figure 7A, 7B and data not shown). The high K^+^-induced Rac membrane association was prevented by inhibiting GGT with GGTi-2147 (Figure 7A, 7B). This result suggests neuronal depolarization may also activate GGT in cerebeller neurons. Next, we determined the effect of high K^+ ^on dendritic development of PCs. We found that high K^+ ^treatment caused a remarkably increase in the number of dendritic branches indicated by number of crossings (Figure 7C-E), as well as dendrite length (Figure 7F and 7G). However, the effect of high K^+ ^was attenuated in neurons transfected with GGTβ-siRNA (Figure 7C-G). The reduction of high K^+^-induced dendrite development caused by GGT-siRNA was exemplified by the decreased number of crossings at the 75 μm circle (Figure 7E) and total dendritic length between 50 and 75 μm circles to the soma (Figure 7G). These results suggest that GGT is required for depolarization-induced dendrite development of PCs.

## Discussion

During cerebellar development, Purkinje cells (PCs) undergo dendrite extension and become elaborately arborescent, followed by synapse formation between dendrites of PCs and climbing fibers (CF) of inferior olive neurons or parallel fibers (PF) of granue cells (GC) [13]. Thus, dendritic arborization and branching of PCs are essential steps for establishing cerebellar neural circuits. Here we find that GGT is enriched in PCs and required for dendrite development of PCs. Furthermore, neuronal depolarization or BDNF is able to promote PC dendrite development, and these effects depend on GGT expression. Thus GGT plays an important role in cerebellum development.

Previous studies have identified a number of factors that regulate growth and branching of dendritic arbors, including external signals, such as neuronal activity and neurotrophins, and a variety of intracellular mediators, such as Rho family small GTPases [23,24]. The combined actions of multiple factors lead to cytoskeletal reorganization or gene expression required for dendritic growth. Due to the large somata and extensive dendritic trees, cerebellar PC has been a good model to study neuronal morphogenesis. By using organotypic slice culture system, many studies have led to identification of a number of molecules that regulate PC dendrite development [13]. These factors include neurotransmitters and their receptors [25,26], neurotrophic factors [27], steroids [28], thyroid hormone [29,30], neuropeptides [31], kinases [32,33], cytoskeleton regulating proteins [34], and cell adhesion molecules [17].

Previously we have shown that GGT regulates dendritic development of cultured hippocampal neurons [12], where GGT activation by BDNF or depolarization increases membrane localization of Rac1 [12], a member of Rho family small GTPases which are important for distinct aspects of dendrite development by modulating actin cytoskeleton [35,36]. It remains to be determined whether Rac1 or other members of Rho family small GTPase also regulate morphogenesis of PCs. In addition to Rho GTPases, dendrite development can be regulated by many other molecules; some of them need to be associated with the plasma membrane in order to be activated efficiently. For example, prenylation of Ca^2+^/calmodulin-dependent protein kinase CLICK-III/CaMKIγ is responsible for its association with the lipid raft and its role in dendritogenesis [37]. In addition to CaMKI, CaMKII also plays important role in dendrite differentiation [38]. Interestingly, inhibition of CaMKII reduced the number of primary dendrites and the total dendritic length of Purkinje cells [33]. It would be of interest to determine the mechanism by which GGT regulates Purkinje cell dendrite development.

Like other cell types, dendrite development of PCs has also been shown to be regulated by neuronal activity [22], and extracellular factors, such as GDNF [27]. In line with this notion, treatment with high K^+^, which is believed to induce depolarization, promoted dendrite development of PCs in cultured cerebellar slices (Figure 7). Consistent with the activation of GGT by high K^+ ^shown in cultured hippocampal neurons, we found that high K^+^-induced dendrite development of PCs was prevented by down-regulation of GGT. A previous report shows that in BDNF-knock out mice, there is a stunted growth of Purkinje cell dendrites [21]. Consistent with this, we found that treatment with BDNF indeed promoted dendrite growth of PCs, although the effect was not as dramatic as predicted (Figure 6). The mild effect of BDNF could be due to the presence of neurotrophic factors in slice culture environment, or the production of BDNF by granule cells [39].

Given that GGT regulate Rac1 activity, which is known to be important for other neural developmental events, such as spine formation [40], it would be of interest to determine the role of GGT in other aspects of cerebellar development. A number of proteins have been shown to be involved in PC dendrite development, including GGT described in this study. Further works are needed to identify the specific regulators for Purkinje cell dendrite development, in particular how elaborate arborization and overwhelmingly dentritic trees are formed.

## Conclusion

GGT is newly identified regulator for Purkinje cell dendrite development and is required for spontaneous, BDNF- or depolarization-induced Purkinje cell dendrite growth and/or arborization. Along with the previous report that shows the role of GGT in dendrite development of cultured hippocampal neurons, the role of GGT can be generalized in various types of neurons.

## Methods

### Reagents, antibodies and plasmids

Recombinant human BDNF was from Peprotech. Spermidine was form sigma. All salts used were from Sigma. Millicells (PICM03050) were from Millipore. Antibodies used for immunostaining were from Santa Cruz Biotechnology (rabbit anti-GGTα), Millipore (rabbit anti-Calbindin), Invitrogen (rabbit anti-GFP, Alexa Flour488 goat anti-rabbit IgG, Alexa Flour 555 goat anti-mouse IgG), Sigma (monoclonal anti-MAP2). Rabbit GGTβ antibody was generated by AbMART using a synthetic peptide derived from GGTβ sequence and affinity purified. The constructs of pSUPER-GGTβ-siRNA, HA-GGTβ and HA-GGTβ^Res ^were described previously [12].

### Rat cerebellar organotypic culture

Cerebellar slices were prepared from P11 SD rat pups. Animals were anesthetized and decapitated. The brains were dissected and sliced in ice-cold EBSS sagittally at the thickness of 400 μm with WPI vibratome. Slices were then transferred onto Millicell and cultured in 5% CO2 at 37°C. The culture medium was modified from that of described previously [14,15]. For BDNF or high K^+ ^treatment, 50 ng/ml BDNF or 10 mM KCl were added into the culture medium 24 h after transfections. The treated cerebellar slices were cultured 3 days in vitro before fixation and image processing.

### Biochemical characterization

Rat brains were homogenized in cold lysis buffer containing 50 mM Tris·HCl, pH 7.5, 150 mM NaCl, 1% Nonidet P-40, 0.5% sodium deoxycholate, and protease inhibitors. After pretreatment with GGTi-2147 (2.5 μM) or DMSO for 45 min, cultured cerebellar slices were treated with KCl (10 mM) or BDNF (50 ng/ml) for 2 hr and lysed. Protein membrane fractionation was conducted by using plasma membrane protein extraction kit (Biovision). Immunoblot follows standard protocol with indicated antibodies.

### Microcarrier preparation and biolistic transfection

Microcarrier preparation was performed according to the manufacturer's instructions (BioRad). Briefly, 75 μg of the plasmids mixture (0.75 μg/μl) and 25 mg of gold particles (1.0 μm in diameter) were mixed in 50 mM spermidine (100 μl). Plasmids-particle mixture was precipitated by adding 1 M CaCl_2 _(100 μl) gradually, followed by 3 × washes with ethanol. The plasmids-coated particles were suspended in 3 ml of 15 μg/ml polyvinylpyrrolidone and loaded into tefzel tubing, the tube was then dried and the microcarriers were ready for use. Cultured cerebellar slices were transfected using Helios Gene Gun system after 2 days in vitro. Testing plasmid was mixed with pCAG-EYFP at a ratio of 3:1.

### Tissue processing and immunohistochemistry

P10 SD rats were anesthetized and perfused with 4% paraformaldehyde (PFA, pH7.4). The brains were then dissected and postfixed overnight. The fixed brains were cryoprotected in 30% sucrose solution and sagittally sectioned at the sickness of 30 μm with cryostat microtome. The sectioned brain slices were incubated in 0.3% Triton X-100 for 30 min at room temperature. After blocking with 10% goat serum in PBS at room temperature for 1 h, the slices were incubated in primary antibodies at 4°C overnight. After 3 × washes with PBS, the slices were incubated in corresponding secondary antibodies for 2 h at room temperature, then the slices were mounted.

Cultured cerebellar slices were washed with PBS, and fixed in 4% PFA at room temperature for 30 min. The procedure for the staining of cultured cerebellar slices was similar to that of cryostat microtome sectioned slices.

### Confocal imaging and data analysis

Images were acquired by Zeiss LSM 510 laser scanning confocal microscopy with a 40 × oil immersion objective. The captured neurons were traced using neurolucida software. Sholl analysis was used to analyze the total dendritic length and dendritic arborization. Data analysis was performed using Student's *t*-test. Errors bars in graphs represent SEM.

## Competing interests

The authors declare that they have no competing interests.

## Authors' contributions

KYW and XPZ performed experiments and analyzed data. ZGL designed experiments. KYW and ZGL wrote the manuscript. All authors read and approved the final manuscript.
